# Editorial: Co-design of rehabilitation programming

**DOI:** 10.3389/fresc.2024.1537063

**Published:** 2025-01-06

**Authors:** Janelle Unger, Dalton L. Wolfe, John Bourke, James Middleton

**Affiliations:** ^1^ Faculty of Health Sciences, Western University, London, ON, Canada; ^2^ Parkwood Institute, London, ON, Canada; ^3^ John Walsh Centre for Rehabilitation Research, Northern Sydney Local Health District, St Leonards, NSW, Australia; ^4^ Faculty of Medicine and Health, The Kolling Institute, The University of Sydney, Sydney, NSW, Australia; ^5^ Royal Rehab, Ryde, NSW, Australia; ^6^ State Spinal Cord Injury Service, NSW Agency for Clinical Innovation, St Leonards, NSW, Australia

**Keywords:** person-centered, co-design, rehabilitation, engagement, health services

**Editorial on the Research Topic**
Co-design of rehabilitation programming

Co-design is an approach in health research that incorporates meaningful lived experience engagement across any stage of the research or quality improvement process (1). Engaging with individuals with lived experience can be used to accomplish discrete goals, such as development of technologies, educational materials, or programs, or it can serve process or structural outcomes, such as reducing research waste, enhancing service delivery or improving organizational governance (2). This type of person-centered approach is critical in the field of rehabilitation, due to the unique needs of individuals with varying levels and types of disability experience, as well as differing life situations and environmental contexts. However, much of the existing literature focuses on primary services (2, 3). There is variation in how co-design is defined and implemented (1, 2), and as more literature emerges on this topic in rehabilitation care, it is important to understand the breadth of the field as it continues to grow. We created this Research Topic to showcase current practices and implementation of co-design in rehabilitation research and service delivery, and to encourage further reflection on how the field can move forward in a socially responsible and impactful way.

This Research Topic consists of 10 articles, each describing important aspects of co-design approaches and how they can be applied in a rehabilitation setting. Among the articles, a common theme was using co-design as a method of identifying factors that may influence the success of various rehabilitation programs, educational tools, or technologies. Studies included multiple and diverse stakeholder groups in their research, demonstrating the importance of using a comprehensive approach to gather perspectives.

A common theme among the original research and methods papers was the use of co-design in working towards discrete outcomes rather than impacting service delivery or governance. Reitzel et al. used a co-design approach that included caregivers, clinicians, and healthcare managers and discussed innovative solutions to enhance access and engagement in pediatric telerehabilitation. Through these discussions, they found that communication, consistency and connection were key factors that could enhance engagement in pediatric telerehabilitation and reduce barriers to care (Reitzel et al.). Shi et al. used one-on-one interviews with a variety of stakeholders within a community-based SCI organization and a rehabilitation center to identify barriers and facilitators as well as collaboration processes to delivering a peer mentorship program for people with SCI; they identified 10 factors that could influence a program's success. Eggiman-Ketter et al. used a similar approach to identify enablers and barriers to implementing an interdisciplinary experiential learning program for undergraduate and graduate students at a local rehabilitation center, resulting in 15 recommendations for program development. Clanchy et al. used a three-phase approach to prioritize end-user feedback regarding a new rehabilitation device and incorporate it with perspectives from other stakeholders to adapt product development. The authors also provide practical suggestions for other researchers who aim to co-design rehabilitation technologies based on their experiences (Clanchy et al.). Jeyakumaran et al. embedded co-design within the development of a novel assistive device platform and described their successes and lessons learned to inform ongoing and future initiatives. Craven et al. focused on knowledge translation, creating a co-designed podcast as an educational tool to disseminate the findings of a recently developed clinical practice guideline. The methods paper presented by Cimino et al. describes the plan to use co-design to facilitate the development of personalized mobility programming for persons with mobility impairments. Two articles described using co-design to address organizational structures or service delivery as higher-level outcomes. The community case study by Giroux et al. adopted a co-design approach to perform a community-based consensus exercise focused on strategic priorities and future directions for a SCI network in Canada. A brief research report by Seko et al. describes the process to co-design a novel service delivery model to support the transition from pediatric to adult care, highlighting the importance of open communication and iterative program development. Lastly, the perspective article by Bourke et al. shares fundamental principles that are essential to implementing co-design approaches in a meaningful and authentic way. This article leaves readers with the challenge of progressing co-designed research towards co-production, a collaborative approach that centres equitable and ethical practices focused on reflective dialogue (Bourke et al.).

The articles submitted to this topic were focused on the fields of pediatric and spinal cord injury rehabilitation. These populations often require extensive and ongoing rehabilitation care throughout much of their life, emphasizing the need for care to be person-centered. A systematic review found that co-design approaches are most often described in the fields of mental health, primary care, and pediatrics, and that each field is distinctive and will benefit from different implementation strategies (2). Lived experience engagement and integrated knowledge translation are also at the forefront in spinal cord injury research and practice, resulting in the recent formation of groups that are focused on bringing together people with lived experience, clinicians, and researchers to bring about change (4). Knowing that co-design approaches are impactful in any population, we encourage rehabilitation researchers and decision-makers to consider how they can best use co-design to support each unique population they support.

The articles in this research topic highlight the importance of co-design methods in developing and evaluating rehabilitation programs, educational tools, and technologies. Using a co-design approach ensures rehabilitation practices are person-centered and are meeting the needs of key stakeholders, including patients, caregivers, and clinicians, as well addressing enablers and barriers within the healthcare system. It is imperative that co-design approaches are carried out intentionally and with people with lived experience at the core, in order for the field to move forwards toward co-production. As the articles in this Research Topic primarily focused on initial development of rehabilitation programs, educational tools, and technologies, it will be important for future research to use co-design to evaluate progress and person-centred outcomes, as well as to reflect on larger impacts in service delivery models and organizational structures, which typically require a higher level of engagement.
